# Multimodal Topical Formulations Combining Synthetic Anti-Inflammatory Agents, Levofloxacin, and Plant Extracts for Veterinary Wound and Inflammation Care: In Vivo Efficacy

**DOI:** 10.3390/vetsci13040399

**Published:** 2026-04-19

**Authors:** Maria-Teodora Pițuru, Marina Ionela Nedea, Miruna Maria Apetroaei-Leucă, Dana Tăpăloagă, Andreea Letiția Arsene, Denisa Ioana Udeanu, Cosmin Șonea, Bruno Ștefan Velescu, Tudor Ion Năstasescu, Constantin Vlăgioiu

**Affiliations:** 1 Faculty of Veterinary Medicine, University of Agronomic Sciences and Veterinary Medicine of Bucharest, 105 Splaiul Independentei, District 5, 050097 Bucharest, Romania; maria.pituru@fmvb.usamv.ro (M.-T.P.); dana.tapaloaga@fmvb.usamv.ro (D.T.); cosmin.sonea@fmvb.usamv.ro (C.Ș.); constantin.vlagioiu@fmvb.usamv.ro (C.V.); 2 Faculty of Pharmacy, “Carol Davila” University of Medicine and Pharmacy, 6 Traian Vuia Str., District 2, 020956 Bucharest, Romania; marina.nedea@umfcd.ro (M.I.N.); miruna-maria.apetroaei@drd.umfcd.ro (M.M.A.-L.); andreea.arsene@umfcd.ro (A.L.A.); denisa.udeanu@umfcd.ro (D.I.U.); bruno.velescu@umfcd.ro (B.Ș.V.); 3 Department of Surgery, Tulcea County Emergency Hospital, 30 Iuliu Maniu Str., 820181 Tulcea, Romania

**Keywords:** wound healing, veterinary topical formulations, anti-inflammatory activity, medicinal plant extracts, experimental burn model

## Abstract

Skin wound management in veterinary medicine requires therapies able to control inflammation, limit microbial burden, and support tissue repair. This study evaluated the anti-inflammatory, wound-healing, and immunomodulatory effects of four novel topical formulations combining synthetic anti-inflammatory drugs, antibiotics, and plant extracts in rat experimental models. Burn injury was induced in male Wistar rats for wound-healing assessment, while kaolin- and dextran-induced paw edema models were used to assess anti-inflammatory activity. The tested formulations were meloxicam, dexamethasone, and levofloxacin; thyme extract with meloxicam and dexamethasone; burdock extract with dexamethasone and levofloxacin; and thyme extract combined with burdock extract. Wound evolution was monitored macroscopically, edema was quantified by plethysmometry, and selected inflammatory mediators were measured by immunoassay. In the burn model, the thyme-containing formulation with meloxicam and dexamethasone, and the thyme–burdock formulation, achieved complete wound closure by the end of follow-up, whereas the reference product did not. In the acute inflammation models, all innovative formulations significantly reduced edema at the main early time points compared with the negative control and outperformed the reference product. The thyme–burdock formulation also showed the most favorable immunomodulatory profile, including normalization of interleukin-10 and marked reduction in interleukin-1 beta in both models. These results support the potential of multi-component topical formulations, particularly plant extract-based combinations, as promising candidates for veterinary wound care.

## Simple Summary

Skin wounds in animals are difficult to manage because they are often accompanied by inflammation, pain, delayed tissue repair, and a risk of infection. This study evaluated four new topical formulations designed to improve wound care by combining anti-inflammatory drugs, antimicrobial agents, and medicinal plant extracts. The products were tested in rats using experimental burn wounds and two models of acute paw inflammation. The formulations containing plant extracts showed the most promising effects. In the burn model, formulations containing thyme with meloxicam and dexamethasone, and the combination of thyme with burdock, achieved complete wound closure by the end of the observation period, whereas other groups healed more slowly. In the inflammation models, all new formulations reduced swelling more effectively than the reference product during the early phase after inflammation induction. The formulation containing only thyme and burdock also showed the most favorable effect on selected inflammatory markers, suggesting a balanced anti-inflammatory and healing response. These findings indicate that multi-target topical products combining conventional drugs and plant extracts may offer useful new options for veterinary wound management.

## 1. Introduction

Cutaneous wounds in animals involve tightly coordinated inflammatory responses, are prone to microbial colonization and infection, and depend on effective tissue regeneration to restore barrier function [1,2,3]. Consequently, optimal wound management requires a multimodal therapeutic strategy aimed at controlling excessive or persistent inflammation [4], reducing microbial burden and limiting biofilm-related complications [2,5], and supporting orderly granulation tissue formation, re-epithelialization, and remodeling (cicatrization) [1,6]. In this context, contemporary veterinary wound care increasingly explores synergistic topical approaches that combine anti-inflammatory drugs, antimicrobial agents, and bioactive natural products, often delivered via modern formulation platforms, to improve outcomes in both acute and chronic lesions while minimizing systemic exposure and treatment burden [7,8]. To address current practice needs, four biocompatible topical formulations were developed and characterized [9], each containing a combination of synergistic synthetic anti-inflammatory and antimicrobial agents and/or standardized medicinal plant extracts. Formulation F1 contains meloxicam, dexamethasone, and levofloxacin; F2 includes thyme extract (*Thymus vulgaris* L.), meloxicam, and dexamethasone; F3 comprises burdock extract (*Arctium lappa* L.), dexamethasone, and levofloxacin; and F4 combines thyme and burdock extracts. These formulations aim to leverage the complementary effects of their components, anti-inflammatory, wound-healing, and antimicrobial, to improve the management of skin lesions in veterinary medicine.

The multi-component approach of formulations F1–F4 is designed so that each ingredient enhances the effects of the others, resulting in a therapeutic outcome superior to the individual actions of the components. The underlying rationale for these formulations is the synergistic effect of synthetic anti-inflammatory agents (NSAIDs and a corticosteroid) with adjuvant natural products and/or antibiotics.

First, meloxicam and dexamethasone act at different levels of the inflammatory cascade [10], providing broader anti-inflammatory coverage. Meloxicam inhibits prostaglandin production (mediators of pain and vasodilation in inflammation), whereas dexamethasone suppresses the release of pro-inflammatory cytokines and reduces leukocyte extravasation [11,12]. When combined, they attenuate both the peripheral component of inflammation (pain, edema) and the cellular/immune component [13]. Clinical studies have shown that pairing an NSAID with a corticosteroid yields additional benefits; for example, in patients undergoing dental surgery, pre-emptive co-administration of diclofenac (an NSAID) and dexamethasone significantly reduced postoperative pain intensity and edema compared with NSAID therapy alone [14,15,16]. These findings support the concept that combined NSAID–corticosteroid therapy provides potentiated analgesic and anti-inflammatory effects while potentially allowing lower doses of each agent, thereby reducing the risk of adverse effects such as gastrointestinal toxicity or systemic immunosuppression [17,18,19].

Second, combining synthetic antimicrobials with plant-derived adjuncts can provide complementary or synergistic antiseptic activity and, by enabling lower effective antibiotic exposures at the lesion site, may help limit selection pressure for resistance. Levofloxacin (a fluoroquinolone) exerts rapid bactericidal activity primarily through inhibition of bacterial DNA gyrase and topoisomerase IV. In contrast, thyme (*Thymus vulgaris*) preparations and burdock (*Arctium lappa*) extracts act locally against a broad range of microorganisms via multi-target mechanisms, including membrane permeabilization/disruption and the antioxidant and metal-chelating actions of plant polyphenols, which can interfere with microbial physiology and the inflammatory–oxidative wound microenvironment [20,21,22,23]. Notably, plant–antibiotic synergism has been demonstrated in vitro: in carbapenem-resistant *Acinetobacter baumannii*, adding *T. vulgaris* essential oil (carvacrol-rich) to imipenem reduced the MIC by up to 16-fold, with synergistic interactions (FICI < 0.5) observed across multiple strains in checkerboard testing [24]. Similar potentiation has also been described for carvacrol combined with imipenem against difficult-to-treat Gram-negative pathogens, supporting an adjuvant effect consistent with increased bacterial membrane permeability [25]. Likewise, combining *A. lappa* extract with levofloxacin (F3) may broaden coverage and add ancillary bioactivities (e.g., antioxidant/anti-inflammatory and reported antiviral actions) through the plant’s lignans and polyphenols, complementing the antibiotic’s spectrum [26,27].

The topical use of *Thymus vulgaris* extract has demonstrated broad clinical applicability in the management of dermatological disorders in both human and veterinary medicine. Owing to its well-documented antiseptic properties, thyme-based topical preparations have been used in the treatment of infected skin lesions, while also promoting granulation tissue formation and re-epithelialization [28]. Similarly, the efficacy and safety of topical *Arctium lappa* extract are supported by a growing body of preclinical and clinical evidence. In animal models, burdock-containing topical formulations have been associated with accelerated wound healing, without complications or delays in tissue repair, and with excellent local tolerability [29].

Moreover, topical administration of phytoextracts also offers important pharmacotherapeutic advantages over systemic routes in the management of skin disorders. Local application allows high concentrations of active constituents to be delivered directly to the affected area while minimizing systemic absorption, thereby enhancing local efficacy and reducing the likelihood of systemic adverse effects [30]. In addition, topical delivery enables exploitation of the local synergistic activity of thyme-derived bioactive compounds, including essential oil constituents and natural antioxidants, which can be effectively incorporated into various pharmaceutical dosage forms such as ointments, creams, and hydrophilic gels [31].

The aim of this study was to evaluate the anti-inflammatory, wound-healing, and immunomodulatory effects of four novel topical formulations combining synthetic anti-inflammatory drugs, antibiotics, and plant extracts in experimental models of inflammation and burn injury in rats.

## 2. Materials and Methods

### 2.1. Chemicals, Reagents, Equipment

Dextran 0.6% solution (Dextran 6000, Sigma-Aldrich, St. Luis, MO, USA); kaolin 10% aqueous suspension (Kaolin, Health Chemicals Co., Ltd., Suzhou, China); urethane 13% solution (Urethane, Sigma-Aldrich, St. Luis, MO, USA); physiological saline; diethyl ether (Sigma-Aldrich, St. Luis, MO, USA).

The Reference product was Mibazon^®^ (Antibiotice SA, Iași, Romania) (tetracycline (2.5%) + erythromycin (1.20%) + neomycin (1.20%) + prednisolone (0.06%)). The comparator was selected as a clinically relevant commercial veterinary topical product used for inflamed and/or infected skin lesions.

ELISA kits for TNF-α, IL-1β, IL-2, IL-6, IL-10, and PG_2_F_α_ were purchased from Elabscience Biotechnology Co., Ltd., Wuhan, China. All assays were performed according to the manufacturer’s instructions.

The used equipment was a digital plethysmometer 76-0220 (Harvard Apparatus, Holliston, MA, USA) equipped with a 3 mL cell, ThunderBolt ELISA analyzer (Gold Standard Diagnostics, LLC, Davis, CA, USA), and an Olympus SP-590UZ digital camera (Olympus Corporation, Tokyo, Japan).

### 2.2. Tested Formulations

The test formulations were prepared as follows: F1 contained meloxicam (0.2%), dexamethasone (0.1%), and levofloxacin (0.5%); F2 contained: F1: meloxicam (0.2%), dexamethasone (0.1%), levofloxacin (0.5%); F2 contained *Thymus vulgaris* L. (thyme) extract (0.5%), meloxicam (0.2%), dexamethasone (0.1%); F3 contained *Arctium lappa* L. (burdock) extract (5%), dexamethasone (0.1%), levofloxacin (0.5%); F4 contained *Thymus vulgaris* L. extract (5%), *Arctium lappa* L. extract (0.5%). The test formulations were developed and characterized according to Pituru M et al. [9]. The concentrations were selected during preformulation based on a combination of literature data, expected topical pharmacological relevance, compatibility with the vehicle, preliminary formulation feasibility, and the intention to remain within locally tolerable ranges. The selected concentrations reflected differences in extract characteristics: thyme extract is an essential oil, while burdock extract is a glycerol-alcoholic extract, with potentially sensitive effects of the essential oil [32].

### 2.3. Ethics

All experimental procedures were approved by the Scientific Research Ethics Committee of the “Carol Davila” University of Medicine and Pharmacy, Bucharest (No. 13618, 24 May 2024). Animals were acclimatized for 3 days after transport. Standard rodent chow and water were provided ad libitum. Environmental conditions were controlled throughout the studies (20–22 °C; 35–45% relative humidity) and monitored using a thermo-hygrometer. Sample size was estimated in accordance with 3R principles. For the paw edema study, animals were fasted for 4 h prior to treatment administration.

### 2.4. Burn Induction

#### 2.4.1. Study Design, Group Allocation, and Treatments

Forty-two male Wistar rats (292 ± 14 g) were randomly extracted from the housing cages and randomly allocated into 7 groups (*n* = 6/group) as follows: Group 1—negative control (healthy, uninjured); Group 2—burn control (burn-induced, untreated); Group 3—reference, single topical dose 0.5 mL/animal applied to the lesion; Group 4—F1, single topical dose 0.5 mL/animal applied as in Group 3; Group 5—F2, single topical dose 0.5 mL/animal applied as in Group 3; Group 6—F3, single topical dose 0.5 mL/animal applied as in Group 3; Group 7—F4, single topical dose 0.5 mL/animal applied as in Group 3. Animals in Groups 2–7 were anesthetized with diethyl ether. The volume of 0.5 mL per animal was chosen to ensure complete coverage of the plantar surface of the rat hind paw, which in adult male Wistar rats of comparable weight (200–300 g) has an estimated surface area of approximately 2–3 cm^2^. This volume is consistent with those reported in the topical anti-inflammatory literature for gel and semi-solid formulations applied to the rat paw [33].

Dorsal hair was removed. Burns were induced using a metal device fitted with a 10 mm diameter disc, heated in boiling physiological saline, and applied to the shaved dorsal area for 15 s. This method is intended to assess wound-healing signals under controlled experimental conditions as a single-dose proof-of-concept for the tested formulations.

#### 2.4.2. Macroscopic Wound Monitoring and Measurement

Wound evolution was documented daily by standardized photography using an Olympus SP-590UZ digital camera. Wound diameter was measured for 17 days; at each time point, lesion size was calculated as the mean of the longest and shortest axes of the affected area.

#### 2.4.3. Calculation of Percentage Healing

Percentage healing was calculated as:

$$\%=\frac{D_{xh}-D_{0}}{D_{0}}\times 100$$

where $D_{xh}$ is the wound diameter at the corresponding time point and $D_{0}$ is the initial wound diameter [34].

#### 2.4.4. Histopathological Examination

Following euthanasia, skin tissue samples were collected from the lumbar region and fixed in 10% neutral buffered formalin for 24 h. The tissues were then trimmed into approximately 2 mm thick fragments, placed in histological cassettes, and fixed for an additional 12 h. After fixation, the samples were routinely processed, dehydrated in graded ethanol, cleared, and embedded in paraffin. The paraffin blocks were sectioned at 4–5 μm, mounted on glass slides, and stained with hematoxylin and eosin (H&E). Histopathological assessment was performed using an Olympus BX43 microscope (olympus Corporation, Tokyo, Japan) equipped with a digital camera, under magnifications of 4×, 10×, 20×, and 40×.

### 2.5. Anti-Inflammatory Activity

#### 2.5.1. Study Design and Group Allocation

Ninety-six male Wistar rats (207 ± 32 g) were randomly extracted from the housing cages and randomly allocated into 12 groups (*n* = 8/group) corresponding to two inflammatory stimuli: kaolin-induced paw edema (C) and dextran-induced paw edema (D), as follows: group 1-C/D (negative control) received distilled water by oral gavage (1 mL/100 g body weight); group 2-C/D (reference) received single topical dose 0.5 mL/animal; group 3-C/D received F1, single topical dose 0.5 mL/animal; group 4-C/D received F2, single topical dose 0.5 mL/animal; group 5-C/D: F3, single topical dose 0.5 mL/animal; group 6-C/D received F4, single topical dose 0.5 mL/animal.

Inflammation was induced by intraplantar injection of 0.2 mL of 10% aqueous kaolin suspension into the right hind paw and 0.2 mL of 0.6% aqueous dextran 6000 solution into the right hind paw, respectively.

#### 2.5.2. Topical Application Procedure

For the reference product and all test formulations, 0.5 mL/animal was applied to the surface of the right hind paw in the region of the calcaneus and talus toward the cuboid and navicular bones, using gentle rubbing with the index finger until fully absorbed (minimum 60 rubs), consistent across all topical-treatment groups. This method is intended to assess the anti-inflammatory activity under controlled experimental conditions as a single-dose proof-of-concept for the tested formulations. The volume of 0.5 mL per animal was chosen to ensure complete coverage of the plantar surface of the rat hind paw, which in adult male Wistar rats of comparable weight (200–300 g) has an estimated surface area of approximately 2–3 cm^2^. This volume is consistent with those reported in the topical anti-inflammatory literature for gel and semi-solid formulations applied to the rat paw [33].

#### 2.5.3. Plethysmometric Measurements

Anti-inflammatory activity was assessed by plethysmometry through measurement of right hind paw volume: at baseline (before induction), and at 1, 2, 3, 4, 5, and 24 h after phlogistic agent injection.

#### 2.5.4. Anesthesia

All procedures were performed under urethane anesthesia (13% solution), administered intraperitoneally at 130 mg/kg body weight.

#### 2.5.5. Calculation of Edema Dynamics

The percentage change in paw volume was calculated as:

$$\%=\frac{V_{xh}-V_{0}}{V_{0}}\times 100,$$

where $V_{xh}$ is paw volume at the corresponding time point and $V_{0}$ is baseline paw volume.

### 2.6. Statistical Analysis

Results were expressed as mean ± SD. Normality was assessed using the Kolmogorov–Smirnov test. If the variables were normally distributed, differences between means were evaluated using Student’s *t* test and one-way ANOVA, differences between ranks were analyzed using the nonparametric Mann–Whitney U test and Kruskal–Wallis test. Statistical significance was considered when *p* < 0.05. Analyses were performed in GraphPad Prism v10.0.0.

## 3. Results

### 3.1. Wound Healing Effect

#### 3.1.1. Diameter of Induced Lesions

Table 1 presents the recorded values of the induced lesion diameter for animals in groups 2–7.

Following burn induction, baseline values did not differ significantly between groups (Kruskal–Wallis test, *p* > 0.05). The healing process and reduction in burn diameter differed across groups as follows:

- Group 2: lesion diameter decreased significantly (Mann–Whitney U test, *p* < 0.05) starting on Day 10 of the experiment, with no complete healing by the end of the observation period;
- Group 3: lesion diameter decreased significantly (Mann–Whitney U test, *p* < 0.05) starting on Day 7, with no complete healing by the end of the observation period;
- Group 4: lesion diameter decreased significantly (Mann–Whitney U test, *p* < 0.05) starting on Day 7, with no complete healing by the end of the observation period;
- Group 5: lesion diameter decreased significantly (Mann–Whitney U test, *p* < 0.05) starting on Day 7, and complete healing was observed by the end of the observation period;
- Group 6: lesion diameter decreased significantly (Mann–Whitney U test, *p* < 0.05) starting on Day 3, with no complete healing by the end of the observation period;
- Group 7: lesion diameter decreased significantly (Mann–Whitney U test, *p* < 0.05) starting on Day 3, and complete healing was observed by the end of the observation period.

#### 3.1.2. Healing Process Dynamics

**Figure 1:**
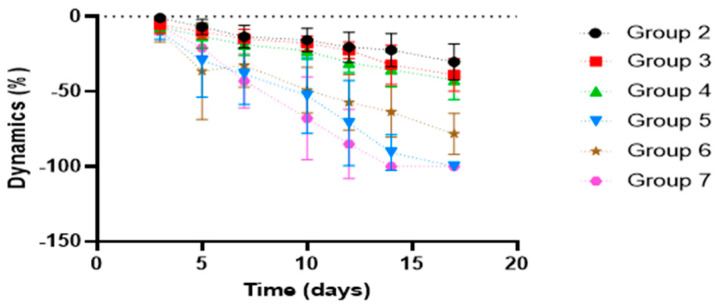
Comparative healing process.

Figure 1 shows, compared with the control group, the dynamics of the healing process, while Table 2 provides a statistical interpretation of the results on the progression of wound healing.

Following statistical analysis of the progression of the kaolin-induced inflammatory process, the overall course of healing differed significantly (Kruskal–Wallis test, *p* < 0.05) across all studied groups compared with the control group (Group 1), suggesting distinct mechanisms modulating the healing process.

The reference product did not significantly affect the dynamics of wound healing compared with the control group. By contrast, formulations F2–F4 displayed a broadly similar pharmacological profile, showing significant wound-healing activity relative to the control starting from Day 3 of treatment (Mann–Whitney U test, *p* < 0.05). Complete closure of the induced burn by the end of the treatment period was observed for formulations F2 and F4.

When compared with the reference product, the therapeutic effect of the innovative formulations F2–F4 was significantly greater from Day 5 of treatment onward (Student’s *t*-test, *p* < 0.05), supporting their superior performance.

#### 3.1.3. Burn Wound Healing Experiment in Wistar Rats

Histopathological examination of H&E-stained skin sections collected after three weeks showed clear qualitative differences between the treated burn groups and the untreated burn control.

**Figure 2:**
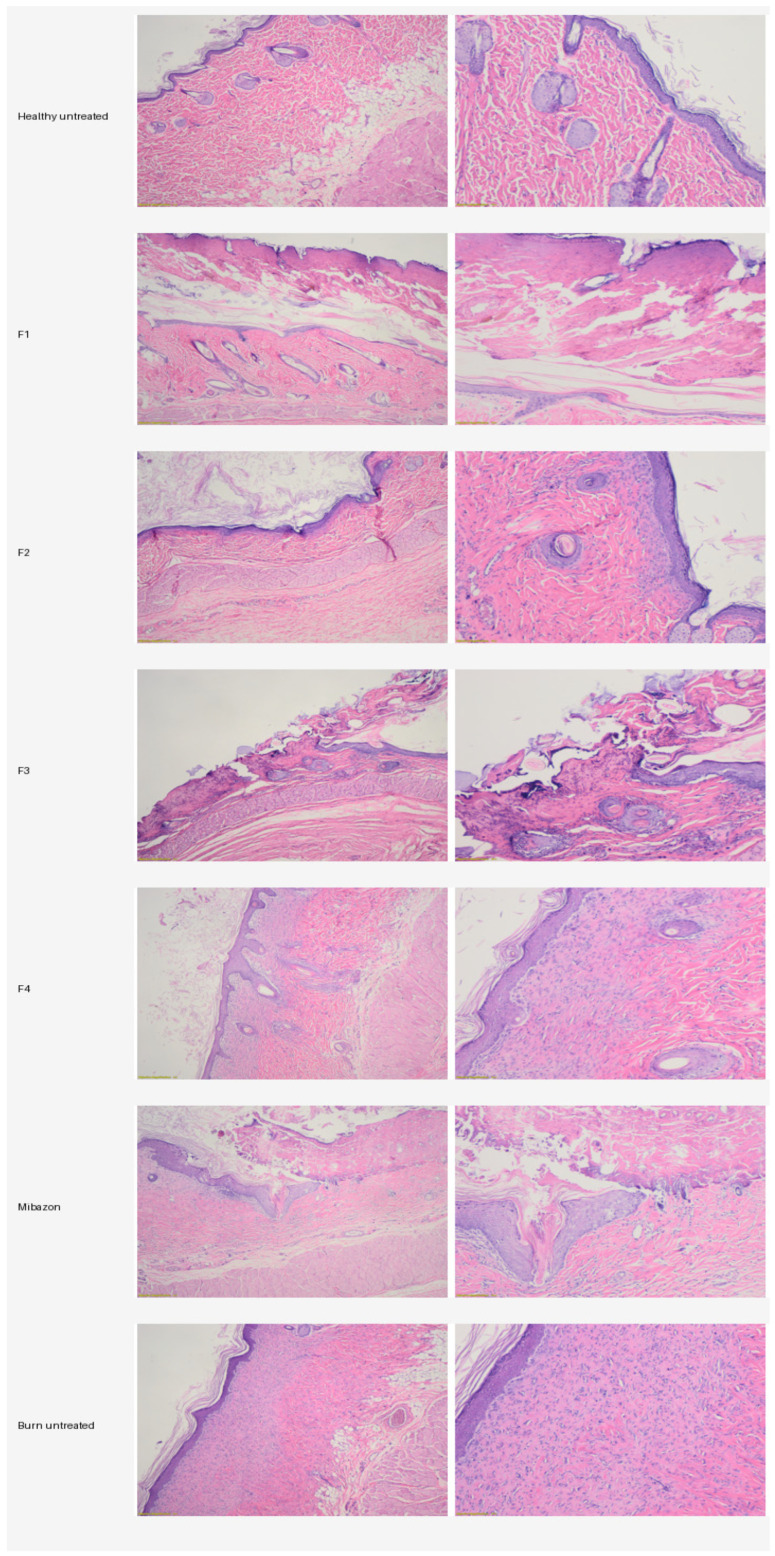
Representative H&E-stained histopathological sections illustrating burn wound healing in experimental groups (F1–F4), reference treatment, untreated burn control, and healthy skin control.

Histopathological examination of H&E-stained skin sections collected three weeks after burn induction revealed qualitative morphological differences between the untreated burn control and the treated groups (Figure 2).

The healthy untreated control displayed the expected normal skin architecture, including a continuous stratified keratinized epidermis, a well-organized dermis, preserved pilosebaceous structures, and no relevant inflammatory changes. This group served as the reference for normal skin morphology.

Group F1 showed morphological features consistent with ongoing repair, including an almost continuous epidermis, mild epidermal hyperplasia, focal hyperkeratosis, moderate dermal fibrosis, and a relatively reduced inflammatory infiltrate composed predominantly of mononuclear cells. Collagen bundles appeared thick and irregularly arranged, suggesting active remodeling, while preserved adnexal structures were still identifiable in some areas.

Group F2 showed an intermediate level of repair. Re-epithelialization was present but incomplete in some fields, where focal ulceration, necrotic tissue debris, and localized inflammatory cell aggregates remained visible. The dermis exhibited dense collagen deposition, persistent fibroblastic activity, and angiogenesis, indicating active tissue repair.

Group F3 exhibited features of ongoing repair, including partial epidermal regeneration, residual inflammatory infiltrate, and focal tissue debris. The dermis contained dense and irregular collagen bundles, active fibroblasts, and newly formed capillaries. Adnexal structures were partially preserved in some sections, indicating residual regenerative potential.

Group F4 showed histological features compatible with a more advanced stage of repair, including a continuous epidermal covering, re-established keratinization, and more regular dermo-epidermal contours. The dermis displayed dense and relatively compact collagen bundles, while the inflammatory infiltrate was limited. Hair follicles and other adnexal structures remained identifiable in several microscopic fields.

The reference-treated group also showed features consistent with progressed healing, including near-complete re-epithelialization, moderate epidermal hyperplasia, dense collagen deposition, mild residual angiogenesis, and limited inflammatory infiltrate. Preserved adnexal structures were visible in several sections.

In contrast, the untreated burn control showed less organized repair. Histologically, the epidermis remained incomplete, the dermo-epidermal interface was irregular, inflammatory infiltrates persisted, and collagen deposition appeared abundant but disorganized, consistent with immature scar formation. Fibroblastic proliferation was more pronounced, while adnexal structures were absent or scarce in affected areas.

Overall, the treated groups showed qualitative histological features compatible with tissue repair, including varying degrees of re-epithelialization, attenuation of inflammatory infiltrate, collagen remodeling, and partial preservation of cutaneous adnexal structures, whereas the untreated burn control retained features suggestive of delayed and less organized healing. These microscopic observations were broadly consistent with the macroscopic wound-healing findings.

### 3.2. Kaolin-Induced Paw Edema

#### 3.2.1. Plethysmometric Results

**Figure 3:**
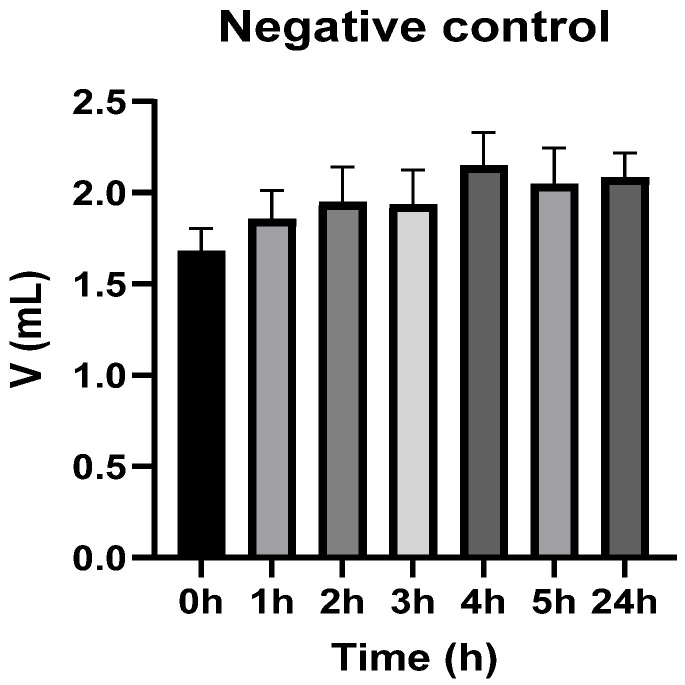
The paw volume in animals (*n* = 8) from group 1-C.

**Figure 4:**
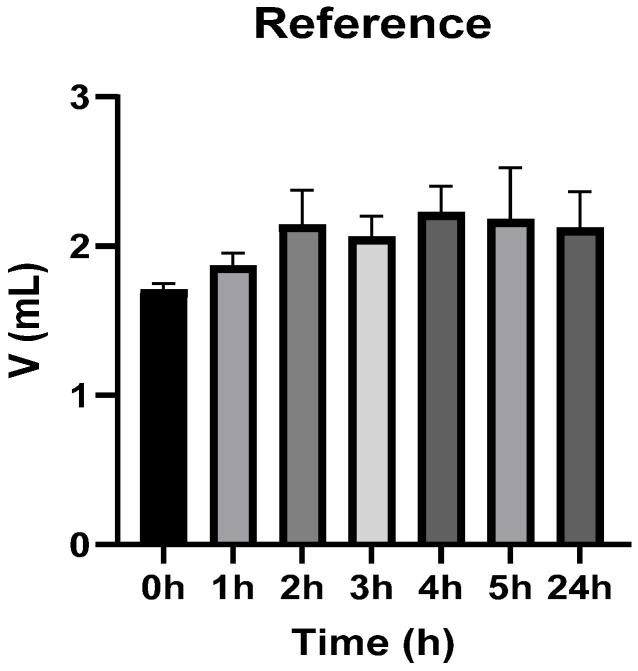
The paw volume in animals (*n* = 8) from group 2-C.

**Figure 5:**
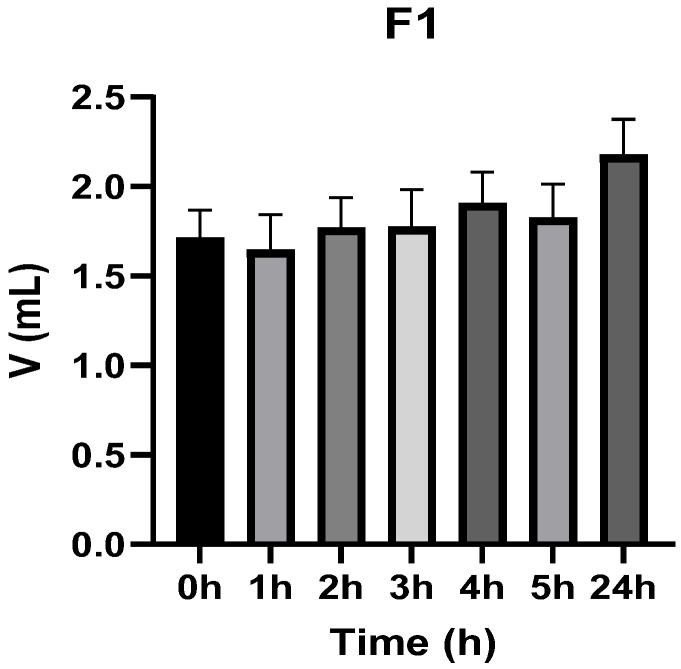
The paw volume in animals (*n* = 8) from group 3-C.

**Figure 6:**
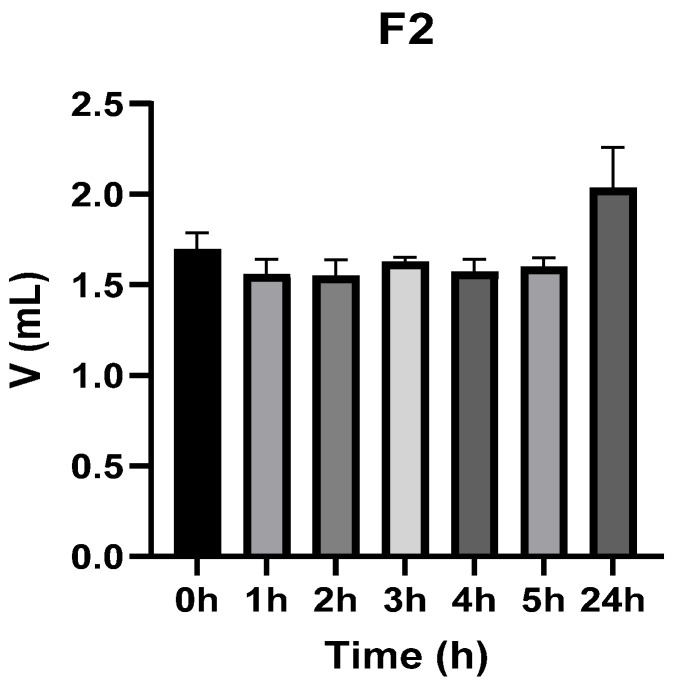
The paw volume in animals (*n* = 8) from group 4-C.

**Figure 7:**
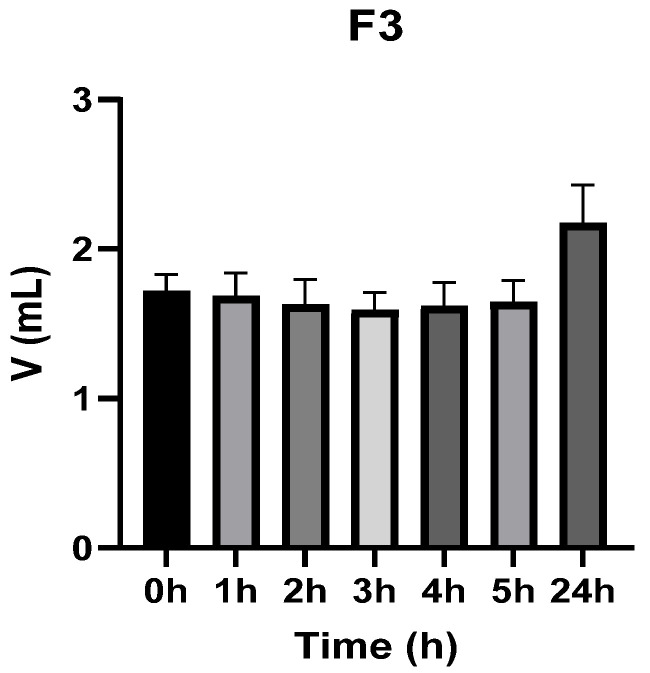
The paw volume in animals (*n* = 8) from group 5-C.

**Figure 8:**
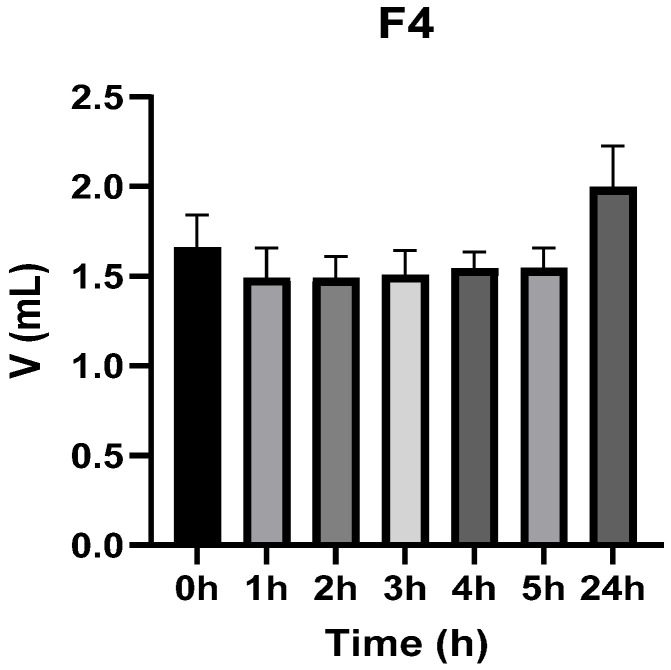
The paw volume in animals (*n* = 8) from group 6-C.

Figure 3, Figure 4, Figure 5, Figure 6, Figure 7 and Figure 8 show the recorded values of kaolin-induced paw edema in animals from groups 1-C to 6-C.

Baseline plantar paw volumes (V0) did not differ significantly among any of the tested groups (one-way ANOVA, *p* > 0.05). Statistical analysis showed that, in Groups 1-C and 2-C, the induced edema values were higher than baseline throughout the entire experiment (Student’s *t* test, *p* < 0.05). In animals treated with F1, induced edema values were not significantly different from baseline at 1, 2, and 3 h after inflammation induction (Student’s *t* test, *p* > 0.05). Animals treated with F2 showed induced edema values comparable to baseline at 3 h (Student’s *t* test, *p* > 0.05). Animals treated with F3 showed induced edema values comparable to baseline at 1, 2, and 5 h (Student’s *t* test, *p* > 0.05). Animals treated with F4 showed induced edema values comparable to baseline at 5 h (Student’s *t* test, *p* > 0.05). Overall, these findings indicate that the innovative gel formulations mitigated the kaolin-induced inflammatory response in the experimental model.

#### 3.2.2. Dynamics of the Inflammatory Process

**Figure 9:**
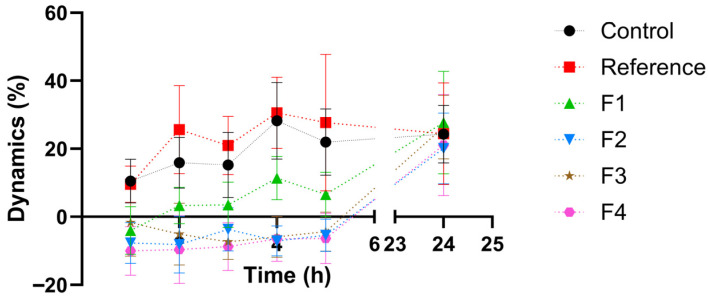
Comparative percentage change in the inflammatory process.

Figure 9 presents, in comparison with the control group, the time-course (dynamics) of the inflammatory response. Table 3 provides the statistical interpretation of the results regarding the evolution of the inflammatory process.

The reference product did not significantly alter the inflammatory response dynamics compared with the negative control group, most likely because formulation-related factors (suspension-type ointment) limited the release of the incorporated steroidal anti-inflammatory agent. In contrast, the tested formulations (F1–F4) exhibited a comparable pharmacological profile and produced a significant anti-inflammatory effect versus the control at all evaluated time points (1, 2, 3, 4, and 5 h post-induction; Student’s *t*-test, *p* < 0.05).

When benchmarked against the reference product, the innovative formulations demonstrated a significantly greater anti-inflammatory activity at each assessment interval (1–5 h post-induction; Student’s *t*-test, *p* < 0.05), supporting their superior therapeutic performance under the experimental conditions.

#### 3.2.3. Inflammation Biomarkers

**Figure 10:**
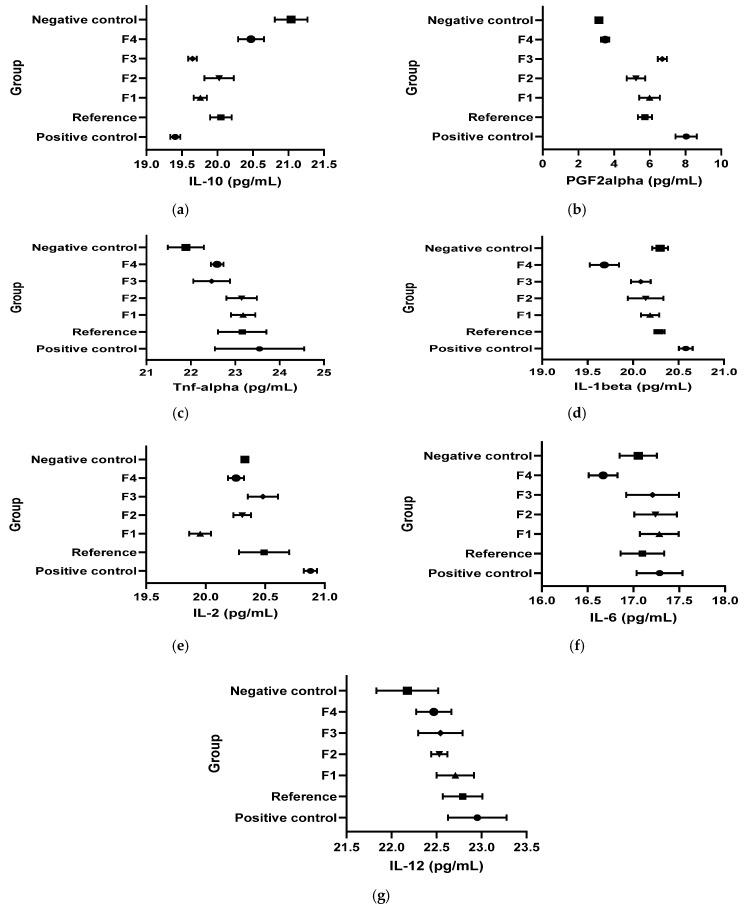
Effects of the tested formulations on inflammatory biomarkers in the kaolin-induced inflammation model. Concentrations of pro-inflammatory mediators were quantified in the kaolin-induced inflammation model for the following experimental groups: positive control, reference, F1, F2, F3, F4, and negative control. The evaluated biomarkers included IL-10 (**a**), PGF2α (**b**), TNF-α (**c**), IL-1β (**d**), IL-2 (**e**), IL-6 (**f**), and IL-12 (**g**), expressed as pg/mL. Data are presented for each group as plotted central values with corresponding error bars, illustrating intergroup variation in cytokine and prostaglandin levels. *n* = 8 animals/group.

Figure 10 and Table 4 present the results obtained for the inflammation biomarkers: interleukins (IL-1β; IL-2; IL-6, IL-10), the cytokine TNF-α and the prostaglandin (PG2Fα) values in the kaolin-induced inflammation model.

IL-10: In the kaolin model, IL-10 was significantly lower in CP, R, and F1–F3 than in CN (Student’s *t* test, *p* < 0.05), while all treatments increased IL-10 versus CP (Student’s *t* test, *p* < 0.05). F4 uniquely normalized IL-10 versus CN (CN vs. F4: n/s; Student’s *t* test, *p* > 0.05 per the narrative phrasing).

PGF2α: Kaolin-induced inflammation significantly increased PGF2α in CP and in several treated groups versus CN (Student’s *t* test, *p* < 0.05 as stated). Compared to CP, R, F1, F2, and F4 were reported to reduce PGF2α (Student’s *t* test, *p* < 0.05), and F4 was described as bringing values close to CN (Student’s *t* test, *p* > 0.05).

TNF-α: TNF-α increased versus CN after kaolin in multiple groups (*p* < 0.05 as stated). However, the document concludes that treatments did not meaningfully affect the TNF-α–mediated inflammatory pathway because TNF-α profiles were similar between experimental groups.

IL-1β: Kaolin increased IL-1β versus CN (Student’s *t* test, *p* < 0.05). R, F1, and F3 decreased IL-1β versus CP (*p* < 0.05) with normalization versus CN (*p* > 0.05). F4 produced a marked decrease below the CN level (*p* < 0.05).

IL-2: IL-2 increased in CP versus CN (Student’s *t* test, *p* < 0.05). F1–F4 reduced IL-2 versus CP (*p* < 0.05), with normalization for F2–F4 (*p* > 0.05 vs. CN) and a decrease below CN for F1 (*p* < 0.05 vs. CN).

IL-6: IL-6 was not influenced overall (*p* > 0.05), but F4 decreased IL-6 versus CN (*p* < 0.05).

IL-12: IL-12 increased in CP versus CN (Student’s *t* test, *p* < 0.05). F2 and F4 decreased IL-12 versus CP (Student’s *t* test, *p* < 0.05) up to normalization.

### 3.3. Dextran-Induced Paw Edema

#### 3.3.1. Plethysmometric Results

**Figure 11:**
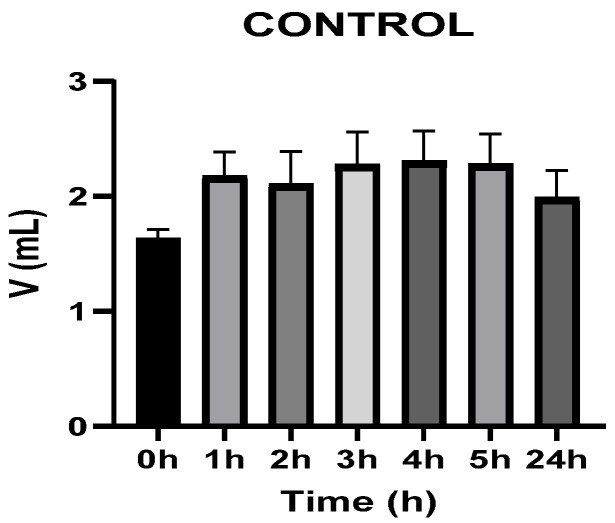
The paw volume in animals (*n* = 8) from group 1-D.

**Figure 12:**
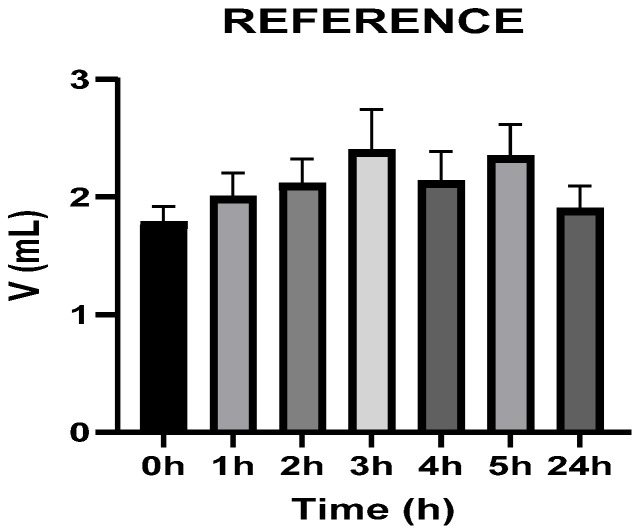
The paw volume in animals (*n* = 8) from group 2-D.

**Figure 13:**
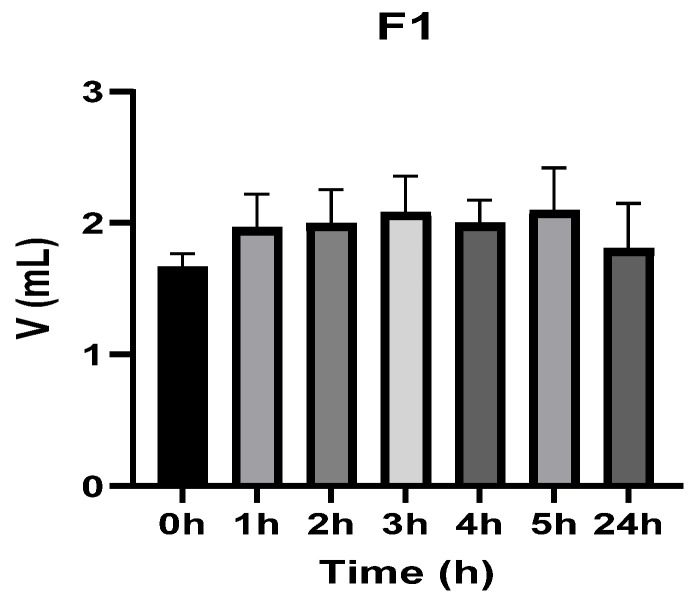
The paw volume in animals (*n* = 8) from group 3-D.

**Figure 14:**
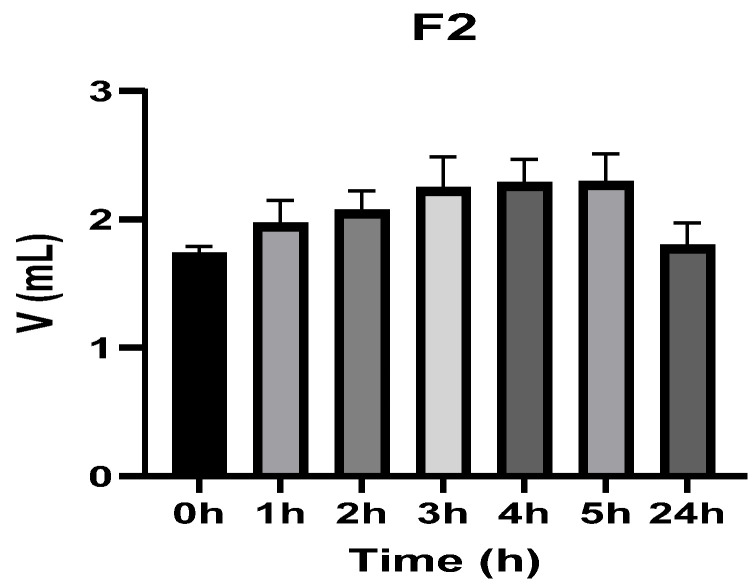
The paw volume in animals (*n* = 8) from group 4-D.

**Figure 15:**
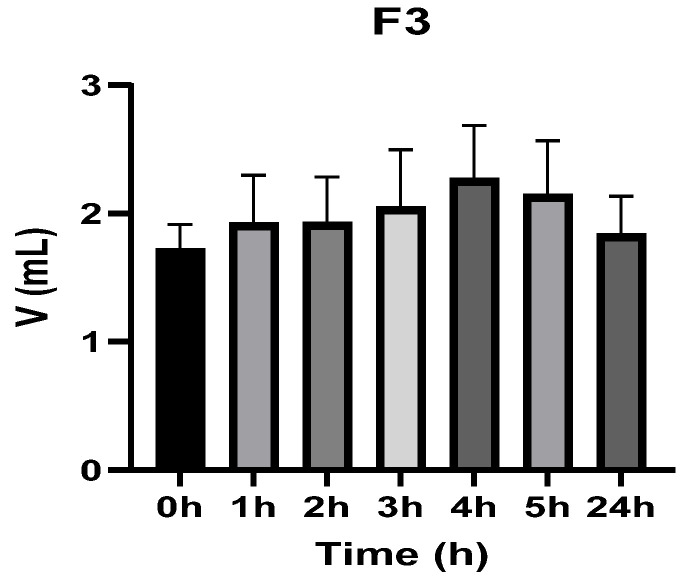
The paw volume in animals (*n* = 8) from group 5-D.

**Figure 16:**
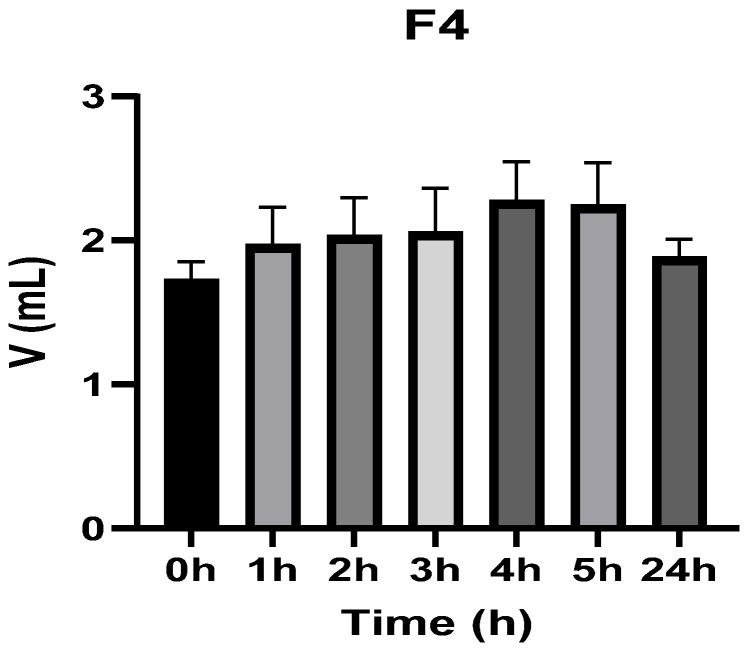
The paw volume in animals (*n* = 8) from group 6-D.

Figure 11, Figure 12, Figure 13, Figure 14, Figure 15 and Figure 16 show the recorded values of kaolin-induced paw edema in animals from groups 1-D to 6-D.

Plant baseline (V0) values for animals in all tested groups showed no statistically significant differences (ANOVA, *p* > 0.05). Statistical analysis indicated that, for groups 1-C and 6-C (treated with F4), the induced edema values were higher than baseline throughout the entire experiment (Student’s *t*-test, *p* < 0.05). Animals treated with the reference, F, F2, and F3, showed induced edema values like baseline at 24 h after inflammation induction (Student’s *t*-test, *p* > 0.05).

#### 3.3.2. Inflammatory Process Dynamics

**Figure 17:**
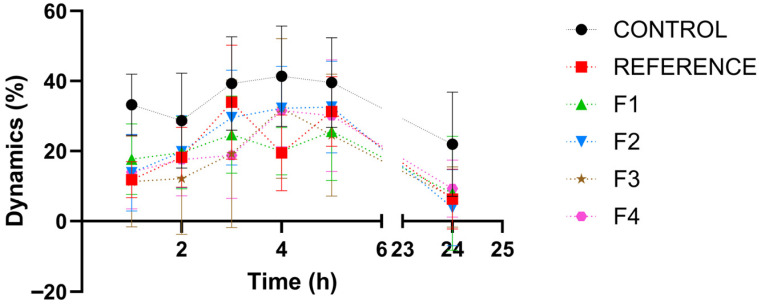
Comparative percentage change in the inflammatory process.

Figure 17 presents, in comparison with the control group, the dynamics of the inflammatory process induced by dextran, while Table 5 provides the statistical interpretation of the results regarding the progression of the inflammatory process.

**Table 5:** Statistical analysis of edema dynamics compared to group 1-D.

Group D vs. Group 1D	Test	1 h	2 h	3 h	4 h	5 h	24 h
Group 2-D	t Student, *p*	n/s	n/s	n/s	n/s	n/s	n/s
	ANOVA, *p*	*p* < 0.05					
Group 3-D	t Student, *p*	*p* < 0.05	*p* < 0.05	*p* < 0.05	*p* < 0.05	*p* < 0.05	n/s
	ANOVA, *p*	*p* < 0.05					
Group 4-D	t Student, *p*	*p* < 0.05	*p* < 0.05	*p* < 0.05	*p* < 0.05	*p* < 0.05	n/s
	ANOVA, *p*	*p* < 0.05					
Group 5-D	t Student, *p*	*p* < 0.05	*p* < 0.05	*p* < 0.05	*p* < 0.05	*p* < 0.05	n/s
	ANOVA, *p*	*p* < 0.05					
Group 6-D	t Student, *p*	*p* < 0.05	*p* < 0.05	*p* < 0.05	*p* < 0.05	*p* < 0.05	n/s
	ANOVA, *p*	*p* < 0.05					

Legend: n/s not significant.

The reference product did not significantly alter the dynamics of the inflammatory response compared with the negative control group. In contrast, the tested formulations (F1–F4) exhibited a comparable pharmacological profile and produced a significant anti-inflammatory effect versus the control at all evaluated time points (1, 2, 3, 4, and 5 h post-induction; Student’s *t*-test, *p* < 0.05).

When benchmarked against the reference product, the innovative formulations demonstrated a significantly anti-inflammatory activity at each assessment interval (1–5 h post-induction; Student’s *t*-test, *p* < 0.05), supporting their therapeutic performance under the experimental conditions.

#### 3.3.3. Inflammation Biomarkers

**Figure 18:**
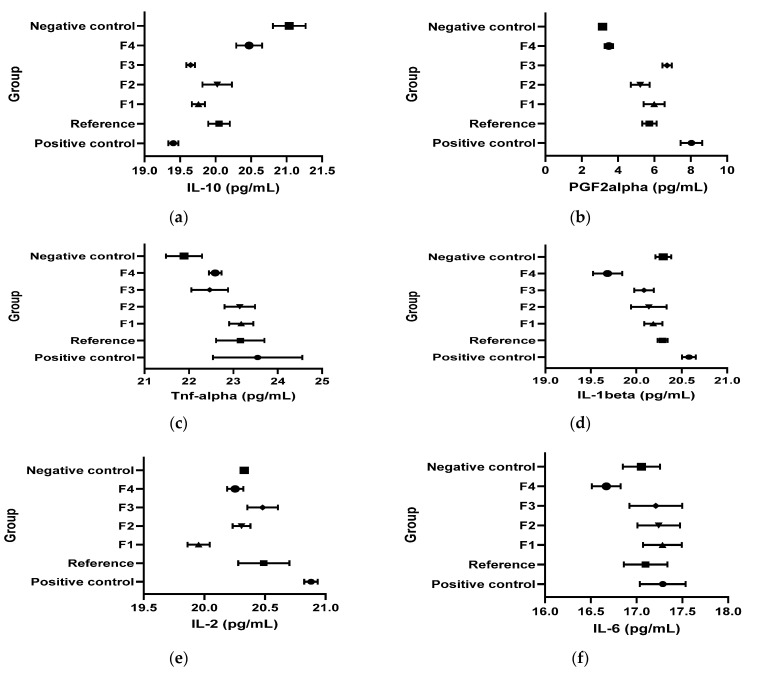

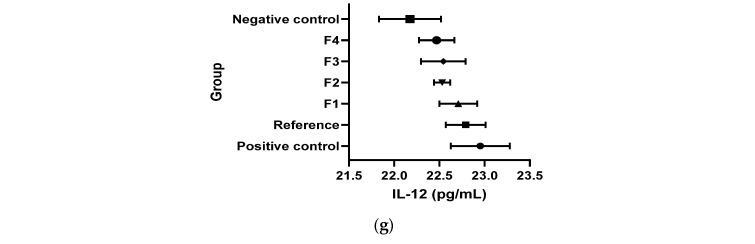
Effects of the tested formulations on inflammatory biomarkers in the dextran-induced inflammation model. Concentrations of pro-inflammatory mediators were quantified in the dextran-induced inflammation model for the following experimental groups: positive control, reference, F1, F2, F3, F4, and negative control. The evaluated biomarkers included IL-10 (**a**), PGF2α (**b**), TNF-α (**c**), IL-1β (**d**), IL-2 (**e**), IL-6 (**f**), and IL-12 (**g**), expressed as pg/mL. Data are presented for each group as plotted central values with corresponding error bars, illustrating intergroup variation in cytokine and prostaglandin levels. *n* = 8 animals/group.

Figure 18 and Table 6 present the results obtained for the inflammation biomarkers: interleukins (IL-10, IL-1β, IL-2, IL-6), the cytokine TNF-α, and prostaglandins (PG2Fα) values in the dextran-induced inflammation model.

IL-10: Dextran reduced IL-10 in CP versus CN (Student’s *t* test, *p* < 0.05). The reference group had IL-10 significantly lower than both CN and CP (*p* < 0.05), while F1–F3 were comparable to CP (*p* > 0.05), and F4 normalized IL-10 versus CN (*p* > 0.05).

PGF2α: Dextran increased PGF2α in CP versus CN (Student’s *t* test, *p* < 0.05), and F2 and F4 were stated to normalize PGF2α (Student’s *t* test, *p* > 0.05).

TNF-α: Dextran increased TNF-α in CP versus CN (Student’s *t* test, *p* < 0.05). The reference normalized TNF-α (Student’s *t* test, *p* > 0.05), while F1–F3 were comparable to CP (Student’s *t* test, *p* > 0.05).

IL-1β: Dextran increased IL-1β in CP versus CN (Student’s *t* test, *p* < 0.05). R and F3 reduced IL-1β versus CP (Student’s *t* test, *p* < 0.05) with normalization versus CN (Student’s *t* test, *p* > 0.05), whereas F4 decreased IL-1β below CN (Student’s *t* test, *p* < 0.05).

IL-2: Dextran increased IL-2 versus CN (Student’s *t* test, *p* < 0.05).

IL-6 and IL-12: No clear effect for IL-6 and no IL-12 pathway mediation in the dextran model.

## 4. Discussion

Kaolin paw edema is a well-established acute inflammation model with a characteristic biphasic time course over the first several hours and is used to discriminate anti-inflammatory pharmacology across mechanistic classes [35,36]. In classic investigations, kaolin-induced edema persisted beyond the first day, and mechanistic probing suggested mediator contributions distinct from purely histamine-driven edema, implicating kinins and other pathways. Dextran-induced paw edema, in contrast, is commonly interpreted as a rapid permeability edema related to mast cell involvement and vasoactive amine release (notably histamine and 5-hydroxytryptamine/serotonin), and it is frequently used to interrogate early-phase anti-edematous mechanisms.

Within this framework, plethysmometry-derived peak separation at 4 h for kaolin is compatible with a later mediator window in which cytokine and eicosanoid regulation becomes more prominent, while dextran shows early timepoint separation across several formulations, consistent with an amine/permeability-dominant early phase [37,38,39,40].

IL-10 is an anti-inflammatory cytokine that constrains excessive innate immune activation, largely via IL-10 receptor–STAT3 signaling and downstream transcriptional programs that suppress the production of pro-inflammatory mediators (including IL-1β, IL-6, TNF-α, and chemokines) in macrophages and related cells [41]. In both models in the uploaded document, F4 is consistently the only formulation reported to normalize IL-10 relative to the healthy control (CN vs. F4: n/s), and it also differs from the inflamed control (CP vs. F4: *p* < 0.05). This pattern supports a mechanistic interpretation in which F4 promotes a pro-resolution or counter-regulatory inflammatory state more effectively than the other formulations, at least as captured by IL-10.

IL-1β is a potent pro-inflammatory cytokine that amplifies local inflammation and can drive secondary mediator cascades [42]. In the present study, F4 produced a marked suppression of IL-1β below healthy baseline in both kaolin and dextran models (CN vs. F4: *p* < 0.05). Taken together, the F4 profile (IL-10 normalization + IL-1β suppression) provides a coherent immunologic explanation for its sustained early inhibition of edema in kaolin (1–5 h significant vs. control) and its time-limited but significant effects in dextran (1 h and 3 h).

Prostaglandins are key lipid mediators of inflammation; while PGE2 is often emphasized, PGF2α is also implicated in acute and chronic inflammatory settings and can engage NF-κB/MAPK-linked signaling depending on tissue context [43]. In both models, F4 was reported to bring PGF2α toward healthy-range values (normalization in the dextran model and near CN in the kaolin model). That observation aligns mechanistically with an anti-edematous phenotype, as prostanoid production is often downstream of inducible cyclooxygenase activity during inflammatory activation [44].

A notable translational signal in the kaolin model is the lack of timepoint-wise significance for the reference product versus the inflamed control, whereas each innovative formulation (F1–F4) differed from the inflamed control at 1–5 h. Across both models, F2–F4 exhibited the most negative Δ% values at the kaolin model’s peak timepoint (4 h: −34,46 to −34,76), suggesting a shared strong ability to blunt the later mediator phase of kaolin edema. By contrast, the dextran model displayed a higher heterogeneity in the timing of the most negative Δ% per group (Figure 16), consistent with an early-phase amine/permeability process where different actives may influence distinct micro-windows of edema formation [45].

Additionally, in the kaolin model, TNF-α was not altered by any treatment, while IL-12 modulation was confined to specific formulations, namely F2 and F4. In dextran, TNF-α normalization was attributed to the reference product, while F4 was explicitly stated not to influence TNF-α signaling. This pattern suggests that in these models, edema reduction (particularly for F4) may be more tightly coupled to IL-10/IL-1β/prostanoid pathways than to TNF-α suppression, at least at the sampled timepoint. The observed wound-healing effects of the tested formulations may be explained by complementary pharmacological mechanisms [33]. In addition, plant-derived components may further support tissue repair by modulating biological processes involved in regeneration, including antioxidant activity, regulation of inflammatory mediators, and stimulation of fibroblast proliferation and collagen deposition [46,47,48].

In this context, formulation F4, containing only *Thymus vulgaris* and *Arctium lappa* extracts, was designed to explore the biological activity of the phytocomplex in the absence of synthetic drugs. The results obtained in the present study support the hypothesis that thyme- and burdock-derived constituents may exert complementary antimicrobial, anti-inflammatory, and tissue-repair activities. Similar concepts have been described for multi-herb preparations, where different phytoconstituents act on complementary biological pathways. For example, a community-based pilot case series reported favorable burn outcomes with a multi-ingredient Burns & Wounds™ ointment containing burdock leaves, suggesting additive or synergistic effects among the phytochemical components [49].

Overall, the investigated formulations (F1–F4) represent a multimodal topical strategy for the management of skin lesions in veterinary patients, combining anti-inflammatory agents, antimicrobial compounds, and phytotherapeutic extracts. Previous studies have demonstrated the veterinary anti-inflammatory and analgesic activity of meloxicam [50], the clinical use of topical glucocorticoids in veterinary dermatology [51], and the feasibility of topical levofloxacin delivery in infected wounds [52]. Furthermore, experimental studies support the antibacterial and wound-healing potential of *Thymus vulgaris* essential oil systems [53], as well as the pharmacological properties of *Arctium lappa* relevant to cutaneous inflammation and tissue repair [54].

These complementary mechanisms may explain the enhanced therapeutic effects observed for some of the investigated formulations, particularly those containing plant extracts, suggesting that multi-target topical therapies may represent a promising approach for improving wound healing outcomes.

*Thymus* spp. and its major constituents, thymol and carvacrol, have been documented to have anti-inflammatory effects in experimental systems, including inhibition of inflammatory edema and leukocyte migration, and reductions in pro-inflammatory mediator production [55,56,57]. Carvacrol has been reported to reduce paw edema induced by histamine or dextran in vivo, directly connecting thyme-derived chemistry to a dextran-relevant mechanistic space [58]. Independently, thyme extracts have been reported to increase IL-10 expression/production in immune-relevant models, which is in line with the present study’s findings regarding F4, which normalized IL-10 [59,60].

Burdock (*Arctium lappa)* bioactivity is often attributed to lignans such as arctiin and arctigenin; arctigenin has been extensively reviewed as an anti-inflammatory compound acting through cytokine modulation and NF-κB/MAPK-associated pathways [61,62,63]. In vivo pharmacology literature reports that arctigenin can reduce paw edema in classic acute models, including significant effects around the 3–4 h window in carrageenan-based paradigms, which is temporally consistent with the kaolin model peak effect at 4 h [64]. Burdock extracts have also been associated with reduced inflammatory cytokines (including IL-1β, TNF-α, and IL-6) in relevant models [65].

Taken together, the literature supports a mechanistic rationale for an extract-only formulation (F4) producing anti-edematous and immunomodulatory effects via (i) dampening of prostanoid biosynthesis/signaling, (ii) suppression of IL-1β-centric inflammatory amplification, and (iii) support of IL-10-mediated counter-regulation [43].

Moreover, the available literature highlights multiple pharmacological effects of *Arctium lappa* extract following topical application, supporting its traditional uses. First, the extract exhibits pronounced anti-inflammatory activity by reducing the production of pro-inflammatory mediators. For instance, both in vitro and in vivo studies have shown that treatment with *A. lappa* extract inhibits COX-2 expression as well as cytokines such as TNF-α and IL-6 at the site of inflammation [66], an effect that correlates with attenuation of local edema and inflammatory response. In addition, burdock demonstrates wound-healing activity by promoting cutaneous tissue regeneration. Daily topical application of an ointment containing burdock extract has been reported to significantly accelerate wound healing and to promote complete re-epithelialization compared with control groups [67]. At the same time, the extract has shown antimicrobial activity against several common cutaneous pathogens. Compounds derived from *A. lappa* have been reported to inhibit the growth of Gram-positive bacteria, such as *Staphylococcus aureus*, Gram-negative bacteria, including *Escherichia coli*, and fungi such as *Candida albicans*, thereby potentially contributing to the prevention of wound superinfection. Finally, burdock also exerts marked antioxidant effects, largely attributed to its high polyphenol content; the extract has been shown to scavenge free radicals and reduce local oxidative stress [68], with beneficial effects on skin repair. Overall, the pharmacological profile of *Arctium lappa* extract includes anti-inflammatory, wound-healing, antimicrobial, antioxidant, antipruritic, and even antiallergic properties, as demonstrated in various in vitro studies and animal models [69,70].

Additional support for the anti-inflammatory potential of thyme essential oil comes from in vivo acute inflammation models. In a mouse carrageenan-induced paw edema assay, oral administration of two regionally distinct thyme essential oils at doses of 100–400 mg/kg produced a dose-dependent anti-inflammatory effect, with the highest dose reducing paw edema at 6 h by 58.4% for one oil and 50.4% for the other. These effects were reported in comparison with diclofenac at 10 mg/kg, supporting the pharmacological relevance of thyme-derived volatile constituents in acute inflammatory conditions. The same study also provided preliminary tolerability data, showing regional differences in acute toxicity, with one oil being toxic at 4500 mg/kg, whereas the other produced no evident signs of toxicity up to 5000 mg/kg [71]. Other experimental studies indicate that thyme essential oil can attenuate inflammatory signaling in innate immune models, with effects frequently aligning with reduced NF-κB-linked cytokine output, decreased COX-2–related transcriptional activity (via carvacrol), and reduced NO production in LPS-activated macrophages. However, the magnitude and even direction of effects can depend on chemotype/harvest stage and formulation [72,73].

### Study Limitations

This study has several limitations that should be acknowledged. The experimental design used a single topical administration, which does not fully reflect routine veterinary practice, where repeated applications are generally required. In addition, the relatively small group sizes may limit statistical robustness. No direct microbiological evaluation was performed. Histopathological assessment was qualitative rather than based on a blinded semiquantitative scoring system. Because the formulations were tested as multicomponent mixtures, the present design does not allow discrimination between the contributions of individual ingredients or between additive and truly synergistic effects. Furthermore, formulation characterization remained limited, as stability and release kinetics were not comprehensively assessed. The study also relied mainly on acute rodent models, without chronic or clinically representative veterinary wound models, which restricts translational relevance. Finally, local and systemic safety were not systematically investigated.

## 5. Conclusions

The tested topical formulations showed anti-inflammatory and wound-healing activity in the used experimental rat models. These findings support further development of multi-component topical therapies for veterinary skin lesion management.

## Figures and Tables

**Table 1:** Induced lesion diameter values (cm).

Group	Day 1	Day 3	Day 5	Day 7	Day 10	Day 12	Day 14	Day 17
2 (burn control)	1.53 ± 0.16	1.51 ± 0.14	1.42 ± 0.13	1.32 ± 0.15	1.28 ± 0.15 *	1.21 ± 0.15 *	1.18 ± 0.12 *	1.05 ± 0.11 *
3 (reference)	1.63 ± 0.15	1.53 ± 0.12	1.46 ± 0.12	1.38 ± 0.13 *	1.33 ± 0.11 *	1.26 ± 0.13 *	1.10 ± 0.21 *	0.99 ± 0.19 *
4 (F1)	1.50 ± 0.16	1.39 ± 0.14	1.30 ± 0.14	1.22 ± 0.15 *	1.16 ± 0.15 *	1.04 ± 0.19 *	0.98 ± 0.23 *	0.87 ± 0.23 *
5 (F2)	1.52 ± 0.21	1.37 ± 0.17	1.09 ± 0.45	0.95 ± 0.36 *	0.73 ± 0.39 *	0.41 ± 0.39 *	0.13 ± 0.17 *	0.00 ± 0.00 *
6 (F3)	1.68 ± 0.19	1.51 ± 0.25 *	1.09 ± 0.57 *	1.14 ± 0.31 *	0.84 ± 0.22 *	0.69 ± 0.28 *	0.59 ± 0.26 *	0.35 ± 0.22 *
7 (F4)	1.53 ± 0.18	1.43 ± 0.20 *	1.21 ± 0.19 *	0.87 ± 0.30 *	0.51 ± 0.47 *	0.25 ± 0.39 *	0.00 ± 0.00 *	0.00 ± 0.00 *

Legend: * Significantly lower compared with Day 1 (the day of burn induction), Mann–Whitney U test, *p* < 0.05.

**Table 2:** Statistical analysis of wound healing treatments.

Group 2 vs. Group	Test	Day 3	Day 5	Day 7	Day 10	Day 12	Day 14	Day 17
Group 3	U Mann–Whitney, *p*	n/s	n/s	n/s	n/s	n/s	n/s	n/s
	Kruskal–Wallis, *p*	*p* < 0.05						
Group 4	U Mann–Whitney, *p*	*p* < 0.05	n/s	n/s	n/s	n/s	n/s	n/s
	Kruskal–Wallis, *p*	*p* < 0.05						
Group 5	U Mann–Whitney, *p*	*p* < 0.05	*p* < 0.05	*p* < 0.05	*p* < 0.05	*p* < 0.05	*p* < 0.05	*p* < 0.05
	Kruskal–Wallis, *p*	*p* < 0.05						
Group 6	U Mann–Whitney, *p*	*p* < 0.05	*p* < 0.05	*p* < 0.05	*p* < 0.05	*p* < 0.05	*p* < 0.05	*p* < 0.05
	Kruskal–Wallis, *p*	*p* < 0.05						
Group 7	U Mann–Whitney, *p*	*p* < 0.05	*p* < 0.05	*p* < 0.05	*p* < 0.05	*p* < 0.05	*p* < 0.05	*p* < 0.05
	Kruskal–Wallis, *p*	*p* < 0.05						

Legend: n/s not significant.

**Table 3:** Statistical analyses of edema dynamics compared to group 1-C.

Group C vs. Group 1C	Test	1 h	2 h	3 h	4 h	5 h	24 h
Group 2-C	t Student, *p*	n/s	n/s	n/s	n/s	n/s	n/s
	ANOVA, *p*	*p* < 0.05					
Group 3-C	t Student, *p*	*p* < 0.05	*p* < 0.05	*p* < 0.05	*p* < 0.05	*p* < 0.05	n/s
	ANOVA, *p*	*p* < 0.05					
Group 4-C	t Student, *p*	*p* < 0.05	*p* < 0.05	*p* < 0.05	*p* < 0.05	*p* < 0.05	n/s
	ANOVA, *p*	*p* < 0.05					
Group 5-C	t Student, *p*	*p* < 0.05	*p* < 0.05	*p* < 0.05	*p* < 0.05	*p* < 0.05	n/s
	ANOVA, *p*	*p* < 0.05					
Group 6-C	t Student, *p*	*p* < 0.05	*p* < 0.05	*p* < 0.05	*p* < 0.05	*p* < 0.05	n/s
	ANOVA, *p*	*p* < 0.05					

Legend: n/s not significant.

**Table 4:** Kaolin-model statistics summary for inflammatory biomarkers.

Marker	NC vs. PC	R vs. PC	F1 vs. PC	F2 vs. PC	F3 vs. PC	F4 vs. PC	R vs. NC	F1 vs. NC	F2 vs. NC	F3 vs. NC	F4 vs. NC
IL-10	*p* < 0.05	*p* < 0.05	*p* < 0.05	*p* < 0.05	*p* < 0.05	*p* < 0.05	*p* < 0.05	*p* < 0.05	*p* < 0.05	*p* < 0.05	n/s
PGF2α	*p* < 0.05	*p* < 0.05	*p* < 0.05	*p* < 0.05	n/s	*p* < 0.05	*p* < 0.05	*p* < 0.05	*p* < 0.05	*p* < 0.05	n/s
TNF-α	*p* < 0.05	n/s	n/s	n/s	n/s	n/s	*p* < 0.05	*p* < 0.05	*p* < 0.05	n/s	*p* < 0.05
IL-1β	*p* < 0.05	*p* < 0.05	*p* < 0.05	n/s	*p* < 0.05	*p* < 0.05	n/s	n/s	n/s	n/s	*p* < 0.05
IL-2	*p* < 0.05	n/s	*p* < 0.05	*p* < 0.05	*p* < 0.05	*p* < 0.05	n/s	*p* < 0.05	n/s	n/s	n/s
IL-6	n/s	n/s	n/s	n/s	n/s	*p* < 0.05	n/s	n/s	n/s	n/s	*p* < 0.05
IL-12	*p* < 0.05	n/s	n/s	*p* < 0.05	n/s	*p* < 0.05	*p* < 0.05	*p* < 0.05	n/s	n/s	n/s

Legend: PC-positive control, NC-negative control, n/s not significant.

**Table 6:** Dextran model ELISA statistics summary (Student’s *t* test).

Marker	NC vs. PC	R vs. PC	F1 vs. PC	F2 vs. PC	F3 vs. PC	F4 vs. PC	R vs. NC	F1 vs. NC	F2 vs. NC	F3 vs. NC	F4 vs. NC
IL-10	*p* < 0.05	n/s	n/s	n/s	n/s	*p* < 0.05	*p* < 0.05	*p* < 0.05	*p* < 0.05	*p* < 0.05	n/s
PGF2α	*p* < 0.05	n/s	n/s	*p* < 0.05	n/s	*p* < 0.05	n/s	n/s	n/s	n/s	n/s
TNF-α	*p* < 0.05	*p* < 0.05	n/s	n/s	n/s	n/s	n/s	*p* < 0.05	*p* < 0.05	*p* < 0.05	n/s
IL-1β	*p* < 0.05	*p* < 0.05	*p* < 0.05	n/s	*p* < 0.05	*p* < 0.05	n/s	n/s	n/s	n/s	*p* < 0.05
IL-2	*p* < 0.05	n/s	n/s	*p* < 0.05	*p* < 0.05	n/s	*p* < 0.05	*p* < 0.05	n/s	n/s	*p* < 0.05
IL-6	n/s	n/s	n/s	n/s	n/s	n/s	n/s	n/s	n/s	*p* < 0.05	n/s
IL-12	n/s	n/s	n/s	n/s	n/s	n/s	n/s	n/s	n/s	n/s	n/s

PC-positive control, NC-negative control, n/s not significant.

## Data Availability

The original contributions presented in this study are included in the article. Further inquiries can be directed to the corresponding author.
